# Supplementary material for: Development and delivery of an allied health team intervention for older adults in the emergency department: A process evaluation
Source: PLoS One. 2022 May 26;17(5):e0269117. doi: 10.1371/journal.pone.0269117 (PMC9135235; doi:10.1371/journal.pone.0269117)
Supplement: S1 File — Detailed narrative analysis of implementation process and delivery. (DOCX) [file pone.0269117.s002.docx]

**Title**: Development and Delivery of an Allied Health Team Intervention for Older Adults in the Emergency Department: A Process Evaluation

**S1 File: Implementation notes**

## Process – Developing the HSCP team intervention in the ED

The process of implementing the HSCP intervention was structured in three main phases.

*Pre-implementation*

In the pre-implementation phase, of approximately 12 months duration, following allocation of funding for the development of the intervention, research work was carried out to establish the evidence that would guide the intervention (i.e., systematic review, participatory study with ED stakeholders, analysis of ED patient flow at the hospital site, ethics approval granted from the Health Service Executive); concurrently, communication activities were undertaken by the academic team to involve relevant stakeholders at the hospital site, including ED medical and nursing staff, hospital Managers and Executives and the Informatics, Planning and Performance Department. These communication activities served to raise awareness about the planned intervention, manage the recruitment of the HSCP team, and identify the structures and supports needed to set up the relevant procedures (e.g., space and equipment in the ED, intervention pathways and inclusion criteria, technical support and infrastructure for data management). As the intervention was to be tested within a randomised controlled trial study design, a research nurse was employed to oversee the research side of the intervention (consenting, data collection at baseline and follow-up, data management and storage).

*Piloting*

In the piloting phase (duration: four-six weeks), after recruitment of the three HSCPs (one Senior Medical Social Worker, one Senior Occupational Therapist and one Senior Physiotherapist) and space allocation in the ED, the team worked to get integrated into the ED environment and identify the most optimal care pathways and criteria, with the support of ED and hospital stakeholders. The team held formal and informal meetings with the ED medical and nursing staff to identify the most optimal ways to capture eligible patients as well as refine the patients’ inclusion criteria and assessment procedures; furthermore, the team liaised with community services to build linkages for the safe discharge home of patients. During this phase, the Department of Informatics, Planning and Performance at the hospital set up the infrastructure, training and support needed for the data collection and management within the trial. As described by the HSCP team, the piloting phase was instrumental not only to know each other and develop an interdisciplinary approach, but also to build trust with other ED staff:

*“It did take a few weeks to get embedded, and then for them to trust us and to get to know us and trust us because it’s the doctors’ signature at the end of the day that sends the patient home. It was the trust. If it wasn’t for that four weeks trial period, as I said, it wouldn’t have had an accurate reflection.”* (HSCP team member)

*Intervention delivery*

The intervention delivery (phase 3), and thus participants’ recruitment, took place from December 2018 until the end of May 2019. The target population for the intervention were frail older adults aged ≥65 years with a variety of index complaints; further details on patients’ inclusion criteria are described in detail in the trial report (1). Eligible patients were approached by the team or the research nurse for recruitment; patients who agreed to take part in the trial were consented, completed a baseline demographic and health assessment and were then randomised to the HSCP intervention or to the control group (i.e., usual ED care carried out by the medical and nursing staff). Once a patient was enrolled in the intervention, the team decided which team member would lead the care based on the patient’s medical conditions. Following the completion of the intervention, the HSCP team was introduced as a permanent service for older adults in the ED.

## Intervention Delivery

*Fidelity*

Considering fidelity, the content of the assessment and the target population were in line with international evidence of ED-based HSCP practice (2), and it was also informed by the national HSCP assessment procedures set up by the Health Service Executive Acute (HSE) Medicine Programme (3). However, as described in the process section above, the team spent time during the piloting period to tailor their procedures based on the feedback provided by existing ED staff members and on the profile of the patient population usually attending the ED where the intervention took place. As recounted by the team, an important adaptation made to the intervention was related to the screening tool used by the team during the patient’s assessment: The team had initially opted for the National Acute Medicine HSCP Common Screening Tool (3) that enabled a detailed description of the patient; however, during the piloting period the ED medical team suggested to refine the screening documentation to highlight the analysis and recommendation made by the team, as this would allow for more timely decisions on the patient.

*Operational matters*

The HSCP team operated Monday to Friday, 8am-5pm, with the three team members starting at staggered times. As part of their daily activities, the first team member to be in the ED in the morning would attend the ED staff handover from night to day shift in order to identify potentially eligible patients that had arrived at the ED outside the team’s operating hours and hadn’t been discharged yet. The triage system was also checked to identify further potentially eligible patients. Furthermore, the team ensured that all the paperwork related to the assessed patients was in order at the end of each day. The team saw on average about two patients per day (Mean=1.82, SD=0.93), ranging from one to five patients. While it was not possible to precisely quantify the duration on the interaction with each patient, the team estimated approximately four-five hours (inclusive of administrative duties), with some time taken by the research nurse (about one hour) to carry out the trial baseline assessment.

*Dose and Reach*

A total of 214 patients were eligible for inclusion in the intervention; of these, 176 accepted to be included, while 38 eligible patients refused to take part because not interested (65%) or in poor physical or mental state (35%). The number of patients seen by the team (n=176) represented 5.27% of all older adults aged ≥65 years who attended the ED during the team operating hours (N=3,337) and 2.27% of all older adults admitted to the ED during the six months of the intervention delivery (N=7,740). The most common index complaints for included patients were limb problems (41.5%), falls (14.7%), unwell adult (13.6%) and back pain (12.5%), with severity ranging from critical (Manchester triage category 2, 14.2%) to urgent (category 3, 79.5%) and standard (category 4, 6.3%).

The team’s operating hours were described by many participants, including the HSCP team, as an important factor of reach. As the intervention went on, it was realised that a lot of eligible patients were GP referrals presenting in the afternoon, but these could not be picked up by the team due to the 5pm finish time. While it was acknowledged that extended working hours might have increased reach, our participants highlighted important contextual barriers to this, including the limits of the scope of practice of allied health professionals, as well as the absence of community services outside normal business hours, which would hinder the ability of a HSCP team to discharge the patient safely:

*“The biggest challenge with that is that if it’s 5pm and you are trying to get information, community services are all gone home. You could get it from family, but to try and get community services might be more difficult. So, it might be as well, if we were to look at that, have access to out-of-hours home help support”* (HSCP team member)

Some participants also mentioned the patient age inclusion criterion (65+) as another important influence on reach. As recounted by the HSCP team, this criterion had been chosen given the focus on frail older adults, who are more likely to be over 65 and thus could benefit the most from the comprehensive assessment provided by a team of healthcare professionals (4,5). Given the complexities of an ED, using an age cut-off was felt as a good trade-off to ensure good patient flow and quality of care. However, it was acknowledged that the criterion may have limited reaching patients younger than 65 years old who would meet other inclusion criteria for the intervention (e.g., index complaint).

Lastly, an important factor of reach and dose was the delivery of the HSCP intervention within a randomised controlled trial, which is discussed in detail in section “Theme 3”.

**References**

1. Cassarino M, Robinson K, Trépel D, O’Shaughnessy Í, Smalle E, White S, et al. Impact of assessment and intervention by a health and social care professional team in the emergency department on the quality, safety, and clinical effectiveness of care for older adults: A randomised controlled trial. PLOS Med [Internet]. 2021 Jul 28 [cited 2021 Aug 30];18(7):e1003711. Available from: https://journals.plos.org/plosmedicine/article?id=10.1371/journal.pmed.1003711

2. Cassarino M, Robinson K, Quinn R, Naddy B, O’Regan A, Ryan D, et al. Impact of early assessment and intervention by teams involving health and social care professionals in the emergency department: A systematic review. PLoS One [Internet]. 2019 Jul 31 [cited 2019 Aug 1];14(7):e0220709. Available from: http://dx.plos.org/10.1371/journal.pone.0220709

3. Royal College of Physicians of Ireland. National Acute Medicine Programme. 2014;(2). Available from: https://www.aoti.ie/attachments/d655de3e-9130-4261-b22f-b78c42b07977.PDF

4. Samaras N, Chevalley T, Samaras D, Gold G. Older Patients in the Emergency Department: A Review. Ann Emerg Med [Internet]. 2010 Sep;56(3):261–9. Available from: http://search.ebscohost.com/login.aspx?direct=true&db=ccm&AN=104920848&site=ehost-live

5. Schnitker L, Martin-Khan M, Beattie E, Gray L. Negative health outcomes and adverse events in older people attending emergency departments: A systematic review. Australas Emerg Nurs J [Internet]. 2011 Aug 1 [cited 2019 Aug 7];14(3):141–62. Available from: https://www.sciencedirect.com/science/article/pii/S1574626711000747
