# Supplementary material for: Development and delivery of an allied health team intervention for older adults in the emergency department: A process evaluation
Source: PLoS One. 2022 May 26;17(5):e0269117. doi: 10.1371/journal.pone.0269117 (PMC9135235; doi:10.1371/journal.pone.0269117)
Supplement: S2 File — List of quotes from each theme. (DOCX) [file pone.0269117.s003.docx]

**Title**: Development and Delivery of an Allied Health Team Intervention for Older Adults in the Emergency Department: A Process Evaluation

**S2 File - Theme Quotes**

| **Theme** | **Quote** |
| --- | --- |
| **1. The team’s motivation and proactive communication promoted acceptability and integration in the ED** | *“They were proactive, already having identified patients and identified issues. They’d be part of handover as well and they have identified people who may need their service. They have already initiated the actual work and then feed back to me, which is fantastic; you are not actually looking for them, they are looking for work and they are initiating it. So, I found them excellent*” (ED nurse 5) |
|  | *“They were very capable of making their presence felt in a positive way and interacting always in a positive manner with the department staff”* (ED doctor 9) |
|  | *“I think it’s a good environment to…what you could do there in four weeks might take you eight to 12 weeks in a ward environment because you have a defined team and a defined closed environment, so it’s very conducive to feedback, communication and testing because there is a real process, a flow there, you can see end to end from triage to once they leave”* (HSCP team member 1) |
|  | *“Keeping key people informed along the way. We had to demonstrate in abundance that we knew what we were doing and were invested in the project. We were open not only for the small things but also significant issues. If we didn’t have that piece, I still think we would have done exactly as we did, it might have been slower to get off the ground.”* (HSCP team member 3) |
| **2. The team’s specialised skills and interdisciplinary approach enhanced patient and staff’s ED experience** | *“We are looking at the same person but with different perspectives, with different methods. And it’s also respecting the other persons in the group and take on board that we all have different ways as long as the assessment is kind of standardised in a way that we are still looking at the same model, and it doesn’t matter how you go about that.”* (HSCP team member 1) |
|  | *“I think, for the patients themselves, whether it was getting a specific intervention, getting community services involved, getting an aid to help them, getting social work stuff happening in the community. I think it’s all very beneficial stuff for patients. And it can be difficult for busy doctors and nurses to sort that out on any given day. Whereas you have somebody dedicated who knows the ins and outs of the community services, knows how to access stuff in a more straightforward manner. It’s much more efficient”* (ED Doctor 2) |
|  | *“There was a lot of pressure to build that trust with the doctors, because it wasn’t the norm and there wasn’t access to it, patients would be sometimes admitted even though there was no need.”* (HSCP team member 2) |
|  | *“Probably a clinical specialist role would have more clinical decision-making powers. As a profession we need to start looking at seeing patient in a primary level, because that’s where’s the real benefit, rather than waiting for the referral. That would free up the medical staff while utilising the hours for the benefit of the patients”* (Hospital staff 2) |
| **3. The project stakeholders’ investment was a key enabler of implementation and acceptability** | *“I was very excited by it, enthused by it. I just thought for me it was a case of ‘we need to have these disciplines in there; if I can use research to prove how effective they are, it’s a win-win’”* (Hospital staff 1) |
|  | *“I suppose, there is always a danger when services are created that they themselves create work. I think that that’s a positive thing in that circumstance, but I suppose we always have a little fear that our community-based colleagues might become aware that the service is available within the ED for specific cohorts of patients and then decide to send patients to the ED because they know that this is available”* (ED doctor 10) |
|  | *“There never has been that collaboration between this kind of hospital side of it and the academics side, let alone also bringing in kind of the IT element of it as well. So, I think there is a good bit of learning around that alright in terms of how we can all play together nicely”* (Hospital staff 3) |
| **4. Using a trial format promoted credibility but caused frustration among patients and staff** | “*Providing information to the patient, going through all the background information with them, giving them time to read through it and understand and then going back and ask whether they consented or not. That was something that I suppose added a bit of time to the assessment. That kind of delayed things a bit*” (HSCP team member 1) |
|  | *“I think that it can be very hard to convince health care management people to fund a new service in the current language in the absence of definitive evidence. So, I think having this worked particularly well, having a research-funded team coming in and showing benefit, having an impact immediately makes a very positive case for doing further interventions in this manner.”* (ED doctor 8) |
|  | *“It’s very difficult to tell somebody that they were in the control group after they have spent some much time giving you so much information, and they really want support and information about other supports in terms of access and assessment. Some of them became quite distressed at times, when they were in the control group”* (HSCP team member 2) |
