# Supplementary material for: Development and delivery of an allied health team intervention for older adults in the emergency department: A process evaluation
Source: PLoS One. 2022 May 26;17(5):e0269117. doi: 10.1371/journal.pone.0269117 (PMC9135235; doi:10.1371/journal.pone.0269117)
Supplement: Attachment — Submitted filename: Review - Wallis_PONE-D-21-31608 v2.docx [file pone.0269117.s004.docx]

**Plos One Journal article review**

**PONE-D-21-31608**

**Development and Delivery of an Allied Health Team Intervention for Older Adults in the Emergency Department: A Process Evaluation**

**Review Comments to the Author
*Overall comments***

This is a generally well written article that provides a process evaluation related to the introduction of an interdisciplinary team of Health and Social Care Professionals (HSCPs) to an ED in Ireland. While it is not a novel concept to include HSCPs in ED making the model of care work efficiently and effectively is often elusive and more research needs to be undertaken in this space. Process evaluation alongside an RCT is excellent research practice in this space.

***Abstract***

Clear and complete

***Introduction***

This section provides a clear background and rationale to the study.

***Methods***

The method section is generally clear and contains all the relevant information, albeit through reference to two previously published articles. It is disappointing that this process evaluation did not involve patients and/or their carers as most work in this area tries to also present a consumer perspective. However, I understand how difficult this can be with this very vulnerable population.

One aspect I would question, given that much of the data related to interviews/focus groups and was therefore qualitative rather than quantitative data, is the use of the heading “Measures” (line 155). The content of this section includes information about the data collected and how the type of data related to the study objectives. Most data are not measuring something but rather describing and exploring concepts and experiences. I would suggest a change to the heading and table heading.

Under the heading context information is provided about the ED but the context of this implementation study is also about the RCT and the intervention being tested. I suggest providing some information here about the intervention (even though it is detailed in other articles it would help the reader to have some basic information here). Later in the article it says the intervention targeted “frail” (line 227) older adults – there is no indication about how this was assessed prior to participants’ inclusion in the trial. The Trial report states, “Prospective patients who had a Manchester Triage System score (MTS) of 3 to 5 (respectively, urgent, standard, or nonurgent) and who were categorised at triage with limb problems, falls, unwell adult, back pain, urinary problems, or ear/facial problems were invited to take part.” This does not include a frailty assessment. The Rockwood Clinical Frailty Scale was completed after consent was obtained. If a frailty assessment was completed before recruitment as part of a screening process I suggest including this information in this article. This information is important as it allows others to assess the transferability of these findings.

One minor point – “data” is the plural form of the noun “datum” and should be linked with the plural version of the verb e.g. “data were…”. Please change throughout.

***Results***

Again, with qualitative studies a more usual heading is “Findings” as this report is not the result of some experiment.

Minor point – sentence in lines 243-245 may need revision the English expression is poor.

I am concerned that this process evaluation contradicts the trial report in relation to patient consent for the RCT. In this article it is reported that due to the research nurse’s holiday the intervention HSCPs consented trial participants. As this was not reported in the trial results article, and because this could significantly affect how free potential participants might have felt about declining an invitation to participate, I believe this needs further clarification. Specifically, the authors need to indicate that this variation to the protocol was reported to and approved by the ethics committee that gave trial approval.

***Discussion***

The comment in the section on Limitations “Lastly, while every effort was made 530 to ensure rigour, potential respondent and researcher's biases associated with qualitative data 531 collection cannot be entirely excluded.” Does not demonstrate a good understanding of the issues of rigour in qualitative studies. I suggest either including a much more comprehensive statement about how this research demonstrated rigour – or because you don’t have the space – deleting this sentence.

I understand that this intervention was trialled in an ED funded by public funds, however, in my experience a major aspect of the success of such interventions is whether recurrent funding can be accessed once the trial is completed. I am surprised there was no discussion of the issues associated with funding. I note that an economic evaluation was not included in the main trial. I believe a comment about this, at least, in the Discussion or as a recommendation for further study might be useful.

The difficulty with getting such programs funded is that if these complex, vulnerable patients spend less time in ED (which is a great outcome for the individuals) the ED beds rarely stay empty. As a consequence, the cost of providing ED services does not decrease. Rather, issues such as overcrowding, ambulance ramping etc may reduce but rarely cost. Arguing this is an important part of securing long term senior management support and on-going funding. Again, a comment in the discussion might be helpful here.
